# Management of divergent stances as a resource to maintain progressivity and social relationships

**DOI:** 10.3389/fpsyg.2024.1436677

**Published:** 2025-03-12

**Authors:** Aija Logren, Sakari Ilomäki, Johanna Ruusuvuori

**Affiliations:** ^1^Department of Social Sciences, Faculty of Social Sciences and Business Studies, University of Eastern Finland, Kuopio, Finland; ^2^Faculty of Social Sciences, Tampere University, Tampere, Finland

**Keywords:** management of stance, stance-taking, epistemics, deontics, affect, divergence, asymmetry

## Abstract

Previous studies have shown the intersubjective and negotiable nature of stance: interlocutors orient to alignment and adjust their stances to achieve closer alignment. In this article, we study the interplay of three axes of stance—epistemic, deontic and affective stance—and the role their management may have in socially relevant tasks. We describe how the three axes can be simultaneously relevant, taken into account, and dynamically shifted by the participants in a specific sequence of action. The three axes are not always equally aligned or disaligned, but instead divergent: some are aligned at the same time when others are disaligned. Through a case study with two data excerpts, we show how the divergence is an interlocutors’ resource to overcome the disalignment of some of the stances, and to eventually achieve sufficient alignment in order to proceed their activity. Our data are drawn from the institutional context of neurological consultations. We examine the interactants’ stance over longer episodes of talk to illustrate their momentary, multimodal interactional work to display and adjust their stances. The interactants deploy different modalities to address the divergent stances, and further, the multimodal and multifaceted nature of turns enable them to orient to several axes of stance at the same time. Instead of merely taking a stance, the interlocutors *manage their stances*—both in terms of adjusting the alignment and the balance of the different axes—and thus maintain the social relationship between themselves and the progressivity of the ongoing task.

## Introduction

In any human interaction, people orient to maintaining their shared understanding about the world (Clark, 1996). This involves paying attention to three omnipresent axes of meaning making that are relevant in creating and maintaining this ‘common ground’: knowledge, power and emotion, i.e., the epistemic, deontic and affective orders of interaction (Stevanovic and Peräkylä, 2014). These three axes become more or less salient in the ways in which we project and recognize different actions in interacting with each other, depending on the type of action that is ongoing (Levinson, 2012). In constructing an action the interactants bring forward their *stance* toward the object or topic in focus. In this article, we describe, firstly, the dynamics of simultaneous, yet potentially divergent epistemic, deontic and affective stances and secondly, introduce a theoretical concept, *management of stances,* to illuminate the momentary interactional work interactants do to display and adjust their stances. Our data are drawn from the institutional context of neurological consultations where participants’ differing roles in terms of the institutional task help to highlight the dynamics of this management work.

**Figure 1:**
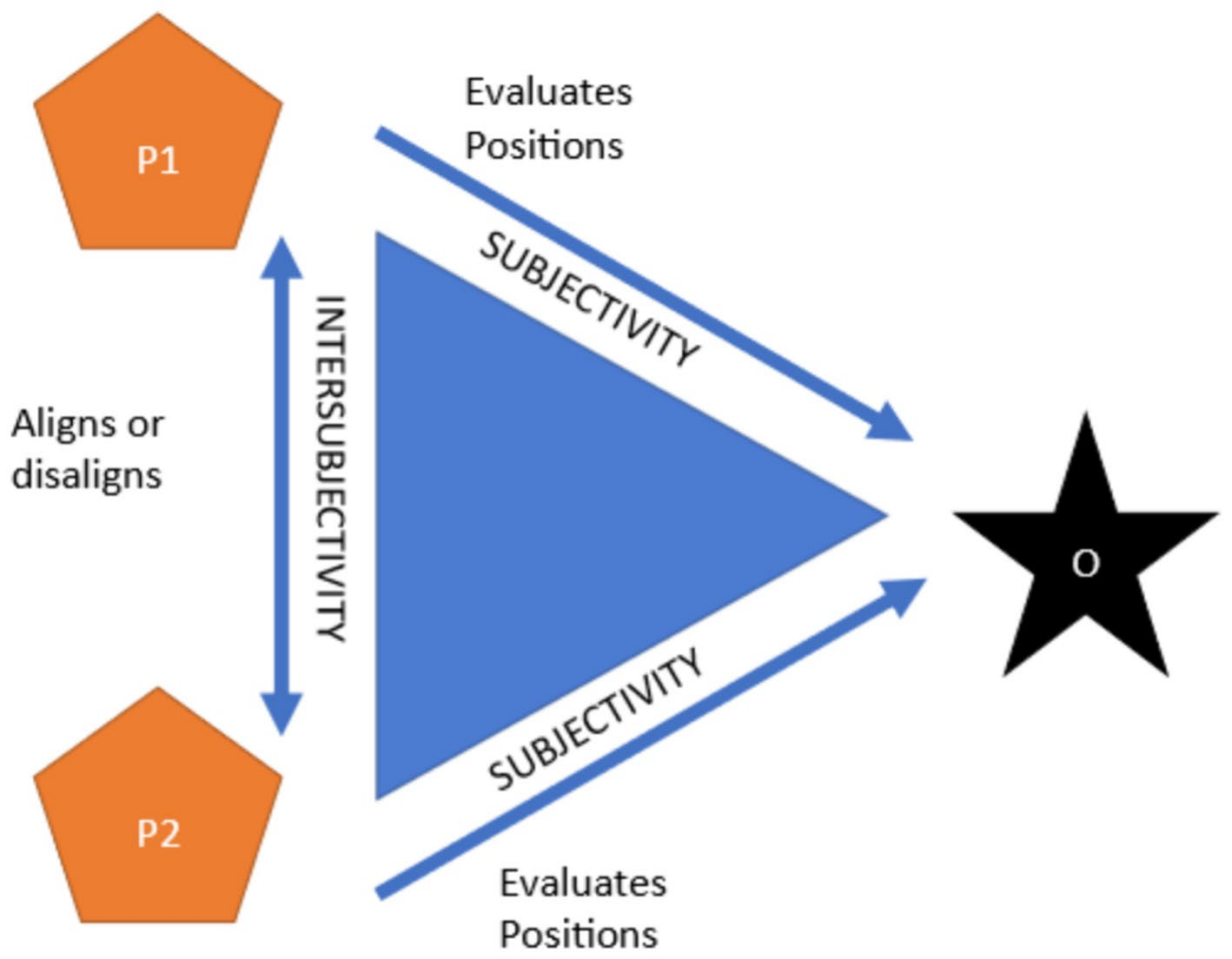
The Stance Triangle (Du Bois, 2007).

Intersubjectivity of stances has been described with a theoretical framework ‘Stance Triangle’ by Du Bois (2007), which illustrates how stance is not merely individual and subjective evaluation of objects, but an intersubjective process. When taking a stance, a person (P1, see Figure 1) evaluates an object (O), and in so doing, positions themself in relation to that object. In other words, by taking a stance, the person displays that they are, for example, able, entitled or obligated to do so (Heritage, 2012; Stevanovic, 2018; Stevanovic and Peräkylä, 2012; Stivers et al., 2011). Nonetheless, because the stances are not displayed in isolation, the interactants’ responses to P1’s stance-taking contribute to it as well. Interactants’ (P2) responses, which also display evaluation of and positioning to the object, i.e., a stance of their own, are either in alignment to the P1’s stance, or disalign with it (Du Bois, 2007). Alignment and disalignment are consequential for the relationship of the interactants, and thus they may finely adjust their subjective stance displays in their following turns and embodied actions to achieve alignment (see Kaukomaa et al., 2013; Ruusuvuori and Peräkylä, 2009).

**Figure 2:**
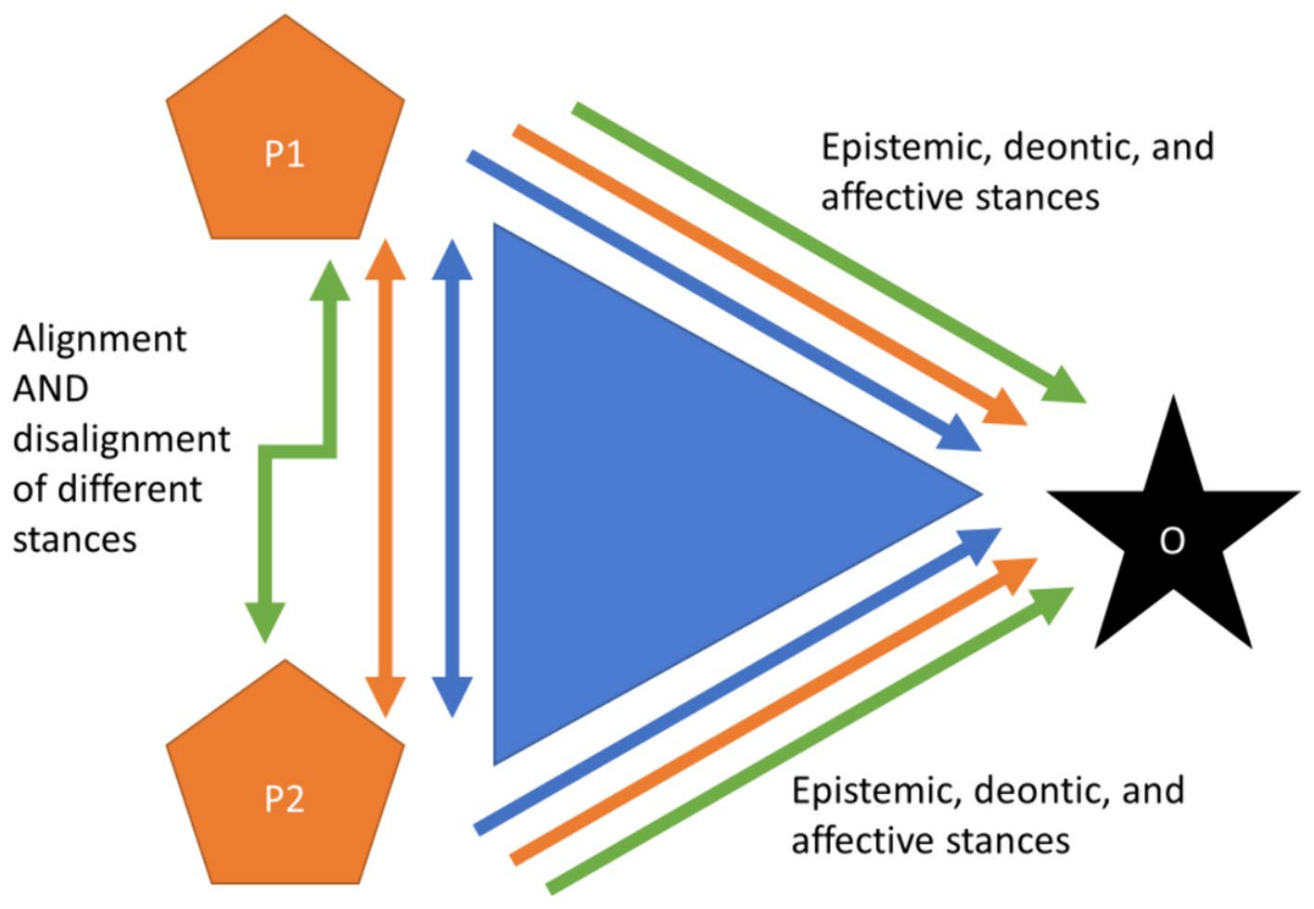
Divergence of stances: simultaneous alignment and disalignment of the different stances within the interlocutors.

We introduce a notion that all three stances—epistemic, deontic and affective stance—can be simultaneously relevant and taken into account by the participants in a specific sequence of action. Furthermore, they may not always be equally aligned or disaligned. Instead, there may be a divergence of the stances, for example the epistemic stance could be disaligned simultaneously when the deontic and affective stances could be aligned (Figure 2).

Because of the possible divergence of stances and their alignment, the fine-grained management is crucial: stances are not static, but they, and perhaps their balance, are finely adjusted and shifted in interaction (Logren et al., 2019, 2020; Ruusuvuori and Peräkylä, 2009). Hence, we argue that the theoretical concept “stance-taking” does not capture the complex and dynamic interactional work participants do in displaying and adjusting their stances and the alignment of the stances. Building on the notions by Küttner (2019), Tai et al. (2022), and Sakita (2013, 2017), who have described how interactants employ specific types of meta-talk to index differences in stances and to negotiate and repair their differences such as disagreement, we suggest that examining how stances are managed over longer episodes of talk better illuminates the intersubjective nature of stance-taking and its social relevance. In this article we illustrate how management of stances is a participants’ resource to maintain progressivity and social relationships.

We present a case study of the divergence of the three stances, and their momentary management through verbal and embodied means in an institutional context, namely clinical consultations. More specifically, drawing from two extracts from neurology outpatient clinics where patients voluntarily bring forward their own understanding about the causes of their symptoms or some suggested treatments, we analyze how the patients display epistemic, deontic and affective stances, how the doctors orient to these different stances in their responses, and how both participants work to adjust the alignment and divergence of their stances.

### Conversation analytic perspective on epistemic, deontic and affective stance

Within our theoretical perspective to the three axes of stance and their alignment and divergence, we adopt the framework of Conversation Analysis (CA) to entangle the momentary sequential work interactants do to display and adjust their stances, participatory roles and preference. This enables us to describe how alignment and divergence of stances is managed turn by turn, and the consequences this may have to the progressivity of the interaction and to the relationships of the interactants. However, in CA alignment has been used also to describe *structural alignment*, that is, how the following turns of talk continue the course of action taken in previous turn (see Stivers et al., 2011). The *alignment of stances* in the sense that has been described in the theory of Stance Triangle (Du Bois, 2007), and thus is the backbone of our study, has been depicted in previous CA studies with concepts such as agreement, acceptance and affiliation. In the following, we review the perspectives to the three axes of stance previous CA studies have to offer, and reflect their relationship to our theoretical framework of the three parallel axes of stance: epistemic, deontic and affective stance—that is, knowledge, power and emotion.

In CA, the knowledge dimension of interaction is referred to as *epistemics* (e.g., Heritage and Raymond, 2005). Management of knowledge in interaction can be differentiated to epistemic stance, which refers to interactional practices through which participants display their evaluation and positioning toward the domain of knowledge, and to epistemic status, i.e., how knowledgeable one participant is entitled to be about some domain of knowledge (Heritage, 2012). Alignment with the previous speaker’s epistemic stance, that is, agreement, is achieved with specific components such as repetition, upgraded evaluations and explicit confirmations, and is often produced with minimal gaps, whereas disalignment, i.e., disagreements are often prefaced, delayed and mitigated (Du Bois, 2007; Heritage and Raymond, 2005, see also Pomerantz, 1984). Furthermore, because of a general preference for agreement in interaction (Sacks, 1987), interlocutors face a practical problem to solve—in Heritage and Raymond’s (2005, p. 15) words: “whose view is the more significant or more authoritative”—whenever they disagree with the previous speaker, i.e., when their epistemic stances disalign. Interlocutors take into account the relationship between their epistemic stance and status, which can be observed in practices such as downgrading and upgrading one’s access to the object of knowledge. For example, questions bring forward some pre-assumptions the questioner has (Heritage, 2009) and turn design and choice of words can be used to convey that some information is known and certain to the recipient—or the opposite (see for example Heritage, 1984, the use of particle “Oh” in English).

The power to make things happen, either in the immediate interactional situation or in the future, is approached in CA through the concept of *deontics* (Ekström and Stevanovic, 2023; Stevanovic, 2018; Stevanovic and Peräkylä, 2012). Deontics refer not only whether an individual gets others to act as they wish but more importantly, how these others consider the individual’s right to imply some future actions in the first place. In many ways, same kind of general principles apply to the management of deontic dimension in interaction as in the case of epistemics. Deontics can be conceptualized through deontic status (i.e., the ability, entitlement or responsibility to make something happen) and deontic stance (i.e., how that is actualized in interaction). Should the recipient affirm the portrayal of the speaker’s deontic right, deontic congruence is formed and vice versa, deontic incongruence exists when the deontic stance is challenged (Stevanovic and Peräkylä, 2012). The production of this (in)congruence is actualized in the alignment or disalignment of interlocutors’ deontic stances, i.e., in acceptance or rejection of previous speaker’s stance. Thus, deontic authority is not a static phenomenon but something that is actualized in interaction: deontic authority varies from domain to domain (that is, a person might have authority about some decisions while not other), and while some status bases for authority may exist, the authority can be resisted in interaction (Stevanovic and Peräkylä, 2012). Disalignment of deontic stances again induces a practical problem for the interlocutors: how to continue, if both parties hold on to their stance. To solve—and to preempt—this dilemma, participants upgrade and downgrade their deontic authority, for example through fitting their demands for action to their entitlement to command and others’ readiness to comply (Antaki and Kent, 2012; Curl and Drew, 2008).

Within the framework of CA, the emotional dimension of interaction becomes salient when the participants orient to some feature of the conversation as affective (Local and Walker, 2008). The affective dimension differs from the epistemic and deontic ones in that it can be (and often is) displayed also, or only non-verbally, with various multimodal means and gestalts. Another difference is that affective status cannot be contested by the other interlocutors—Sacks (1992, Vol II, 242–243) talks about entitlement to experience, meaning that witnessing an event and enjoying or suffering from the experience caused by it, makes a person more entitled to know and tell about it than someone who has only second-hand information about the incident in question. Thus, notwithstanding potential differences in epistemic or deontic status, affective status remains with the person who ‘owns the experience’ (term coined by Peräkylä and Silverman, 1991). Affective stance, then, refers to the affective evaluations that are displayed in interaction and that display the interlocutors’ emotional orientation (Goodwin et al., 2012). Although emotion may not be made lexically explicit in conversation, it is omnipresent in the sense that any act in interaction may be interpreted as displaying some affective stance. For example, eye-rolls are interpreted as displaying dissent toward the immediately preceding action (Clift, 2021), and turn-initial frowns foreshadow complications within the upcoming story-telling (Kaukomaa et al., 2013). Even the lack of showing affect when it would be relevant is also a display of an affective stance: for example, failing to validate other interlocutor’s troubles-telling may signal lack of empathy and lead to pursuing validation (Ruusuvuori, 2005). Alignment or disalignment (i.e., affiliation or disaffiliation) of affective stances may be less crucial to the progressivity of the ongoing interaction as affective stance displays are often non-verbal and do not necessarily follow the sequential order of verbal interaction (cf. Mondada, 2018). Thus, while affective stances may be reciprocated (as in validating a troubles-telling), they may also be left unnoticed or untopicalized (as in responding to troubles-telling with advice (see Jefferson and Lee, 1992). Nevertheless, disalignment may have consequences for the social relationship of the interlocutors (Du Bois and Kärkkäinen, 2012).

Analytic interest has typically focused on one axis at a time (which is reflected also in the differing terminology described above), but our initial empirical observation has been that interlocutors can orient to all three at the same time, and moreover, from different perspectives at the same time. Therefore, it is necessary to examine, firstly, *how* they accomplish this complex, threefold orientation: what kind of features of turns enable the three stances to intertwine, and how interlocutors deploy different modalities to address the three axes and their divergence. This is also the reason we have chosen the analytical concept *alignment*, which depicts the parallel and intertwined relationship of the three stances. Secondly, *why* this threefold orientation seems to be relevant for the participants? One reason for this may lie in the context of the interaction. The roles and relationships of the participants may be particularly salient in specific types of contexts, e.g., in formal, hierarchical institutional contexts such as clinical encounters. Thus, the epistemic, deontic and affective statuses and managing the three stances becomes specifically relevant for the interlocutors. Furthermore, the tasks of the encounter the participants pursue may increase the importance of the progressivity of the encounter.

### Clinical encounters as a context for managing stances

Clinical encounters form a specific kind of institutional and interactional context, which shapes the production of all three, epistemic, deontic and affective aspects in interaction: We analyze cases from neurology consultations, as with the institutional task of finding proper medical treatment for the patient, and the pre-assigned expertise of the professionals, there is a clear asymmetry between the rights and obligations of the participants. This means that the participants’ epistemic and deontic statuses remain stable as compared to everyday conversation. This also means that patients may have to do additional interactional work to make their own concerns heard, which then makes negotiation over disaligned stances relevant (for overviews on institutional interaction, see Drew and Heritage, 1992; Heritage and Clayman, 2010).

Regarding the epistemics, there is a basic orientation to two domains of knowledge and their primary epistemic authorities: the medical knowledge, for which the professional is the authority, and the patient’s lifeworld knowledge, which includes for example bodily sensations, earlier experiences with treatments, and preferences about different solutions. While these domains exist, their boundaries are not strict in a sense that for example the patient could not express their understanding about the causes of symptoms. Instead (Curl and Drew, 2008) when patients produce their ideas, they balance between commitment to the idea and demanding an uptake from the professional (Gill, 1998; Gill and Maynard, 2006). Thus, both patients and professionals generally produce turns that are in congruence with their epistemic status: patients by lowering their epistemic stance (Gill and Maynard, 2006) and professionals by maintaining theirs (e.g., Peräkylä, 1998, see Stivers et al., 2011 on epistemic (in)congruence).

The clinical setting provides structure for the management of deontics, regarding both progression within the consultation (e.g., Robinson and Stivers, 2001) and decision-making (Land et al., 2017). While only the professional has the right to prescribe treatments, the patient always has the option not to comply, and for example refrain from taking the prescribed medicine. Thus, some kind of display of commitment is crucial when participants make decisions in clinical interaction (Stivers, 2006). Due to the institutional context where doctors have the deontic rights to prescribe medication, patients’ ways to resist the decision are usually subtle. Patients may, for instance, ask questions on possible other medication or treatment, or refrain from committing to the treatment suggestion by mere nods (Stivers, 2002). They may also resort to their undeniable epistemic rights to know more about their current symptoms.

In clinical interaction, as well as undoubtedly in many other institutional contexts, affective stance is subordinate to epistemic and deontic stances which are focal in regards fulfilling the institutional purpose of the encounter. For example, while the patients may produce affective turns which in everyday conversation would make relevant an equally affective response, in clinical context it is often relevant to maintain the orientation to the task (Jefferson and Lee, 1992). However, the affective axis may still be oriented to as relevant, especially by the patients who may at times pursue validation to their troublesome experiences treating the professional’s task-oriented response as insufficient (Ruusuvuori, 2005, 2007). Furthermore, while epistemic and deontic authority may become a subject of contest, affective status cannot be contested in the same way by the other interlocutors—instead, the patient is treated as entitled to their own experience (Peräkylä and Silverman, 1991). Thus, while patients’ deontic status in deciding which treatment is appropriate is low as compared to the doctor, they may resort to their affective experiences in resisting the doctor’s decisions [as in resisting the doctor’s proposal to go to hospital by claiming to be afraid of hospitals, (see Ruusuvuori, 2005)].

As this last notion demonstrates, epistemics, deontics and affect are not separate entities, and it has been suggested that especially the first two intertwine in clinical interaction (Heritage, 2013; Stevanovic, 2018; Stevanovic and Peräkylä, 2012). For example, patients can treat medical professionals’ assertions about available treatments either as suggestions or as mere informings (Toerien, 2018) and when acceptance of a suggestion is expected, patients’ questions can be treated as a way of doing rejection (Stivers, 2005). Thus, whether and how epistemics, deontics or affect are relevant for the participants must be determined *in situ*. However, while the relationship between epistemics and deontics has gained interest, especially from the viewpoint on how epistemic authority can be used to build deontic authority (see, e.g., Peräkylä, 1998) and how knowledge configures in decision-making (see, e.g., Toerien, 2018), the management of all these three dimensions simultaneously has received less attention (see however, Koskinen and Stevanovic, 2022; Logren, 2019; Logren et al., 2019; Petraki and Clark, 2016 on how epistemic and affective stance intertwine). In this article, we examine how the three stances can have parallel relevance for the interactants, and how some of the different stances may be simultaneously aligned and some disaligned, that is, divergent.

## Materials and methods

The data are drawn from a corpus of 86 neurology outpatient clinic consultations, collected in 2021–2024 (data collection ongoing) in two hospitals. The participants are patients who are visiting neurology clinics for diagnosis or treatment adjustments and their doctors and nurses. The data are in Finnish. The consultations were video-recorded and transcribed using Jeffersonian transcript symbols (Jefferson, 2004) that were augmented to depict multimodal actions (Mondada, 2001, see Supplementary material for transcription symbols and original Finnish transcripts).

During unmotivated looking at the data, we recognized those patients’ tuns, in which they put forward their understanding and ideas about the causes of some symptoms or treatments to be interesting interactional situations regarding how the shared understanding and the alignment of epistemic stances is achieved step by step. In parallel with analyses concentrating on epistemics, we recognized how in some cases also deontic and/or affective stances were made relevant. We then concentrated on the cases where multiple stances are made relevant. Finally, we selected two contrasting cases for the analysis of this article to showcase the complexity of managing three stances simultaneously: one depicting a simple case focusing only on epistemics, and one with all three stances being made relevant (see Schegloff, 1987, on conversation analytic approach to single-case analysis).

The study is part of “Reliable Knowledge for Health Care: Process and Practice of Shared Decision Making” research project, funded by the Strategic Research Council (SRC) established within the Academy of Finland (project numbers 31213358415 and 31213584181). Before the data collection, the research project obtained ethical approval from the local ethics committee for medical research in Pirkanmaa region, Finland (reference code R21057), organizational permissions from the participating hospitals and signed informed consent from the participants. The data extracts are pseudonymized.^1^

## Results

Our results are twofold. On a more theoretical level, we demonstrate how epistemic, deontic and affective stances can co-exist while being divergent—that is, participants’ stances can align on some of the three while simultaneously disalign on other(s). This phenomenon is potentially subordinate to the institutional task at hand. On a more empirical level, we show how different modalities can be used to manage the different stances and their alignment. Furthermore, by achieving alignment in one axis it is possible to mitigate disalignment in the other two. Thus, the management of divergent stances appears to manage the relationship and the progress of the situation.

**Figure 3:**
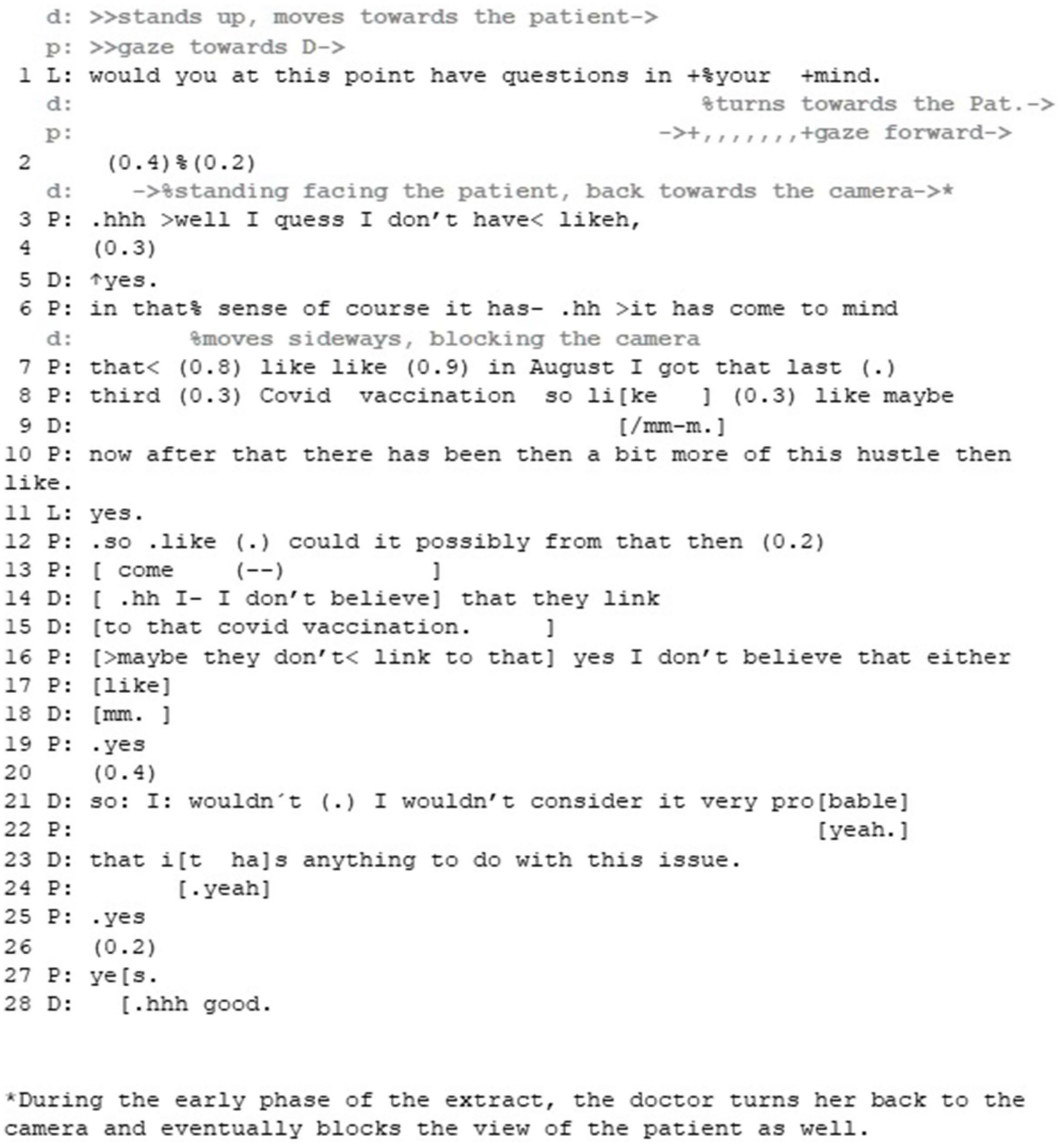
Extract 1—Managing disaligning epistemic stances to achieve closer alignment.

Our first extract depicts a simple case where the participants manage primarily the epistemic axis. The extract is 22 min into the consultation: based on the history-taking the participants have decided that the doctor will consult a colleague to discuss the case before setting a diagnosis. Right before we join the action the doctor has initiated the transition to physical examination (Figure 3).

While the patient initially passes the opportunity to ask a question (lines 1–3), after the doctor’s continuer (line 5) he starts to produce his idea that the vaccination for the SARS-CoV-2 virus could be the reason for his symptoms. The patient frames the idea as something that has occurred to him spontaneously without his own effort (cf. Halkowski, 2006; Ruusuvuori, 2000, line 6), and provides the grounds for the idea by evaluating the potentially close timing of the vaccination and his symptoms (lines 7–10), referring to his symptoms vaguely as “this hustle.” As the doctor merely receives the idea as information with *yes* (line 11) the patient continues by explicating the idea (line 12). The patient brings his idea forward in the form of a question, making the doctor’s uptake relevant. In addition to framing the idea as passively occurring, the question format also enables the patient to bring this idea forward without strong public commitment to it (compared to, for example, stating that he thinks the covid vaccination has caused the increase of the symptoms, cf. Gill, 1998).

Through these practices the patient produces his epistemic status as low and his stance as uncertain. Furthermore, he does not pose the idea straightforwardly but only after passing the first opportunity to ask a question and prefacing it—in short, despite being offered the chance to do it, he seems to treat it as a dispreferred action, and his right to know about it as low.

In her treatment of the patient’s idea, the doctor disagrees with it, but also downgrades her epistemic authority by using the word *believe* (lines 14–15). Thus, while the patient treats the doctor as the more knowledgeable party (from whom it is relevant to ask for information) the doctor partially denies this position. By doing so, she manages to simultaneously appeal to her own professional expertise on one hand, and to present the matter as her personal viewpoint on the other.

Interestingly, the patient produces his third turn to the doctor’s answer in overlap with its ending, potentially expressing affirmation of what the doctor has just said (see Drew, 2009, on the relationship between overlap onset and affirmation). In his turn, the patient agrees with the doctor’s view (that vaccination is not the cause for his symptoms) and recycles the word *believe* as well as the Finnish clitic -*kään*, producing further non-commitment to the idea in a co-operative way (Goodwin, 2013; VISK §839).^2^ After establishing this shared evaluation and positioning toward the idea, the doctor repeats her view (lines 21–23), this time upgrading her epistemic stance by using the expression *consider probable* (in comparison to mere believing) and using the expression *anything to do with it*.

In this example, both interlocutors seem to orient primarily to the epistemic stance, and carefully manage the disalignment of their stances. By mitigating his epistemic status and providing his evaluation of the topic at hand as a vague idea in a question format, the patient seems to pre-emptively prepare his stance as susceptive to change. Even though the doctor disagrees with the patient’s idea on a factual level, the stance itself is designed so that it disaligns as little as possible. The fact that the patient rushes to confirm the doctor’s evaluation of his idea, further works toward alignment between the two. Only after the patient has displayed a stance that abandons the idea, does the doctor strengthen her epistemic stance in declining the idea. By doing so, both participants manage in a stepwise manner to introduce and address a topic that could potentially lead to disalignment, and obtain a shared understanding about it while avoiding the overt disalignment. That is, neither of the interlocutors display a strong stance toward the topic initially but modifies their display of stance in relation to the stance of the other.

**Figure 4:**
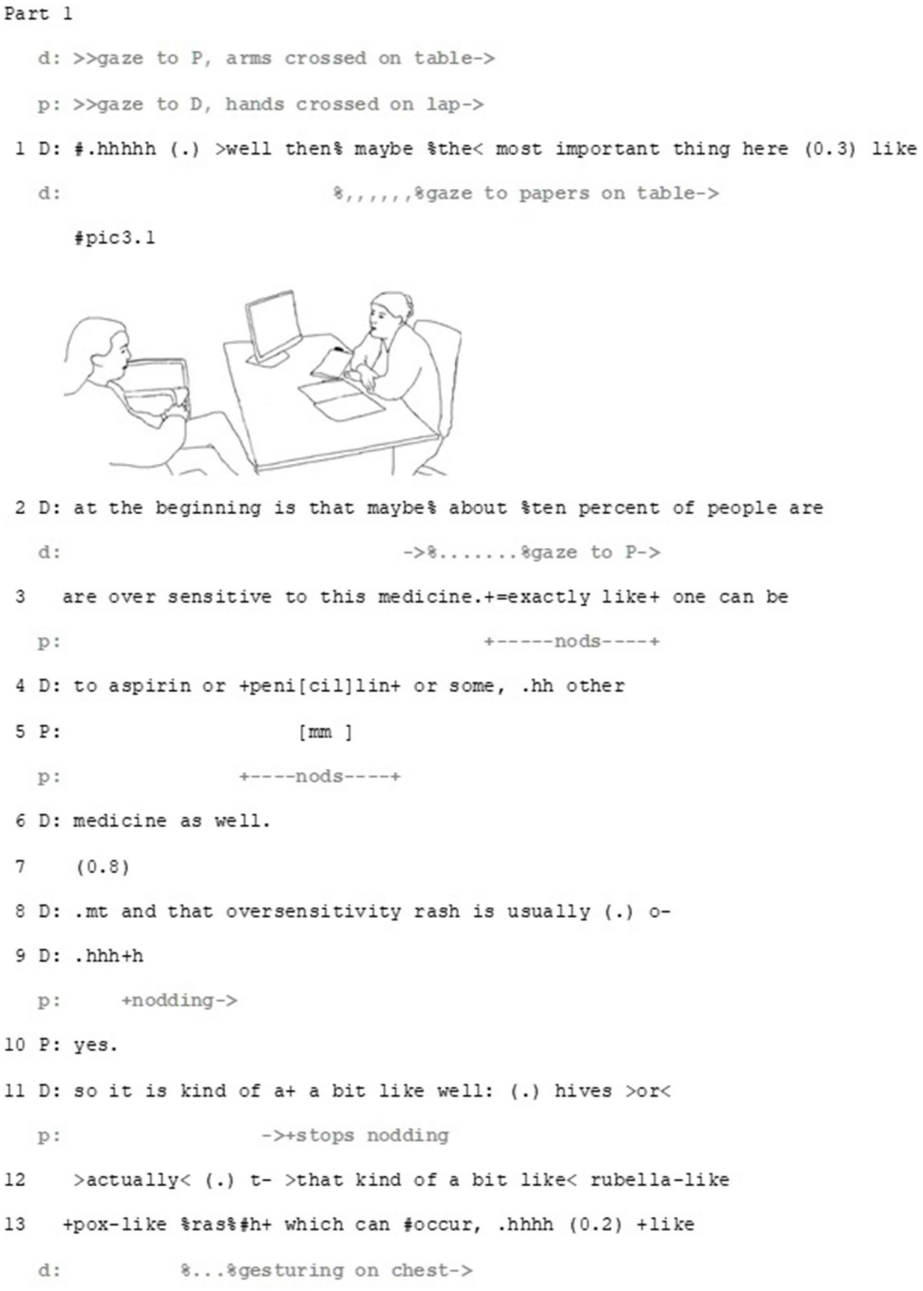
Extract 2—Divergent epistemic, affective and deontic stances and their management—Part 1.

Against this background, the second extract exemplifies how, while displaying an epistemic stance toward an idea, patients can also (and potentially primarily) make deontic and affective stances relevant. To achieve this, the patients can employ different turn-design features, such as extreme-case formulations and bodily imitations. Correspondingly, doctors can manage simultaneous and potentially divergent stances in their responses. We unveil the extract in five parts. The first part takes place when the patient has just received a diagnosis. The doctor has initiated the decision-making sequence with a conditional imperative (*now we should start the medication then*). After the patient’s preliminary acceptance, the doctor has progressed to describe the dosage and the side-effects. We join the action as the doctor has just mentioned the possible changes in the blood counts and starts to describe a more serious side-effect: intolerance to the medicine (Part 1) (Figure 4).

The doctor describes the potential yet unlikely case of drug allergy and provides information that would help the patient to recognize the side effect. This is done by first comparing the medicine at hand to other medicines and penicillin specifically (lines 3–6), and then by providing information about the rash it might cause (lines 8–13), the rash’s potential location (lines 13–15) and the bodily sensations the patient can observe to detect the side effect (lines 17, 19). The depiction is done multimodally, through combination of talk and gesture (pic 3.2 & 3.3). Throughout this segment, the doctor performs one of the basic tasks of the consultation, providing information, thus displaying epistemic expertise. When the doctor vocalizes the word *penicillin* the patient both nods and produces a minimal response (lines 4–5), thus displaying more intense epistemic recognition than earlier during the turn.

**Figure 5:**
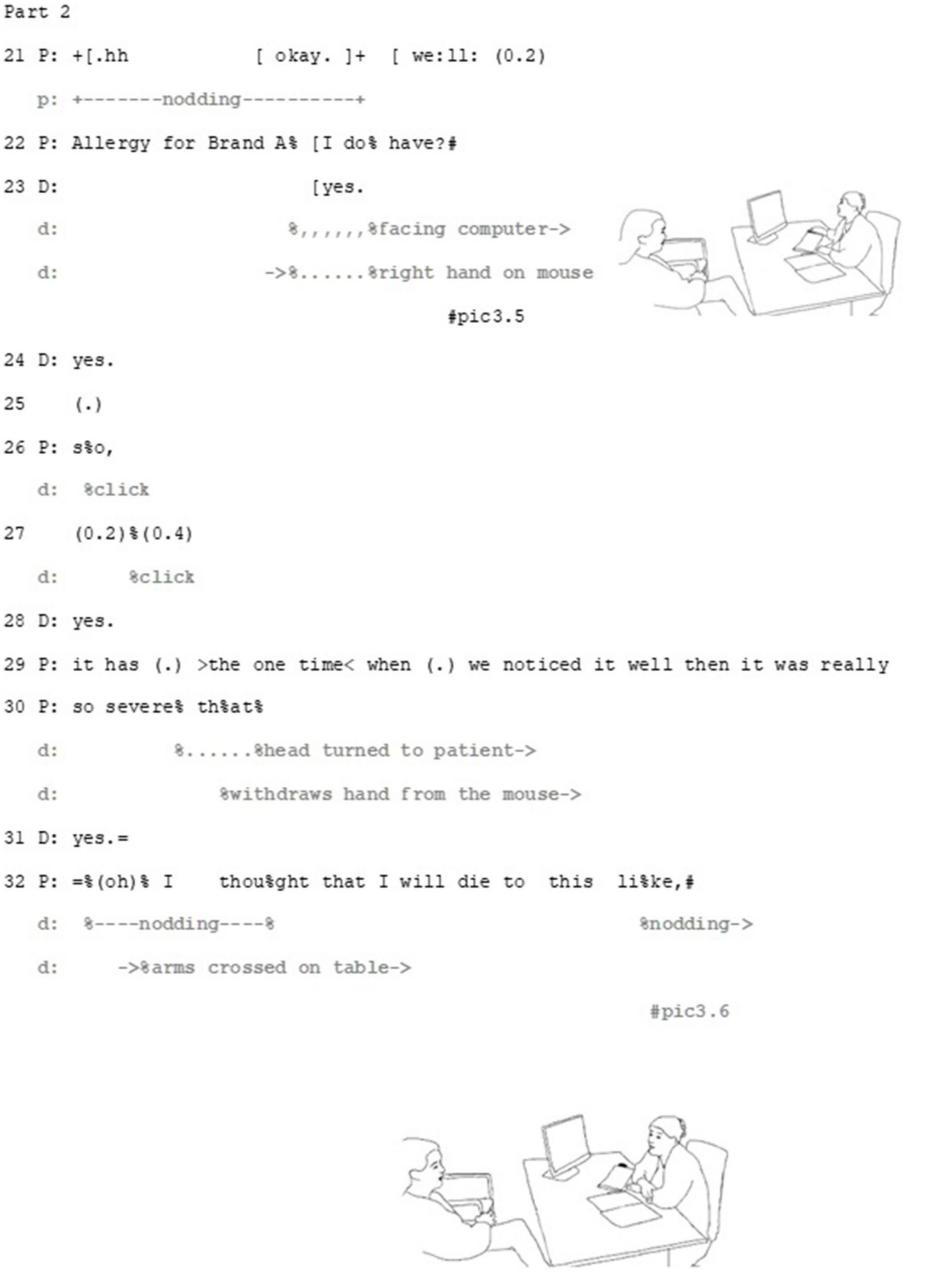
Extract 2—Part 2.

**Figure 6:**
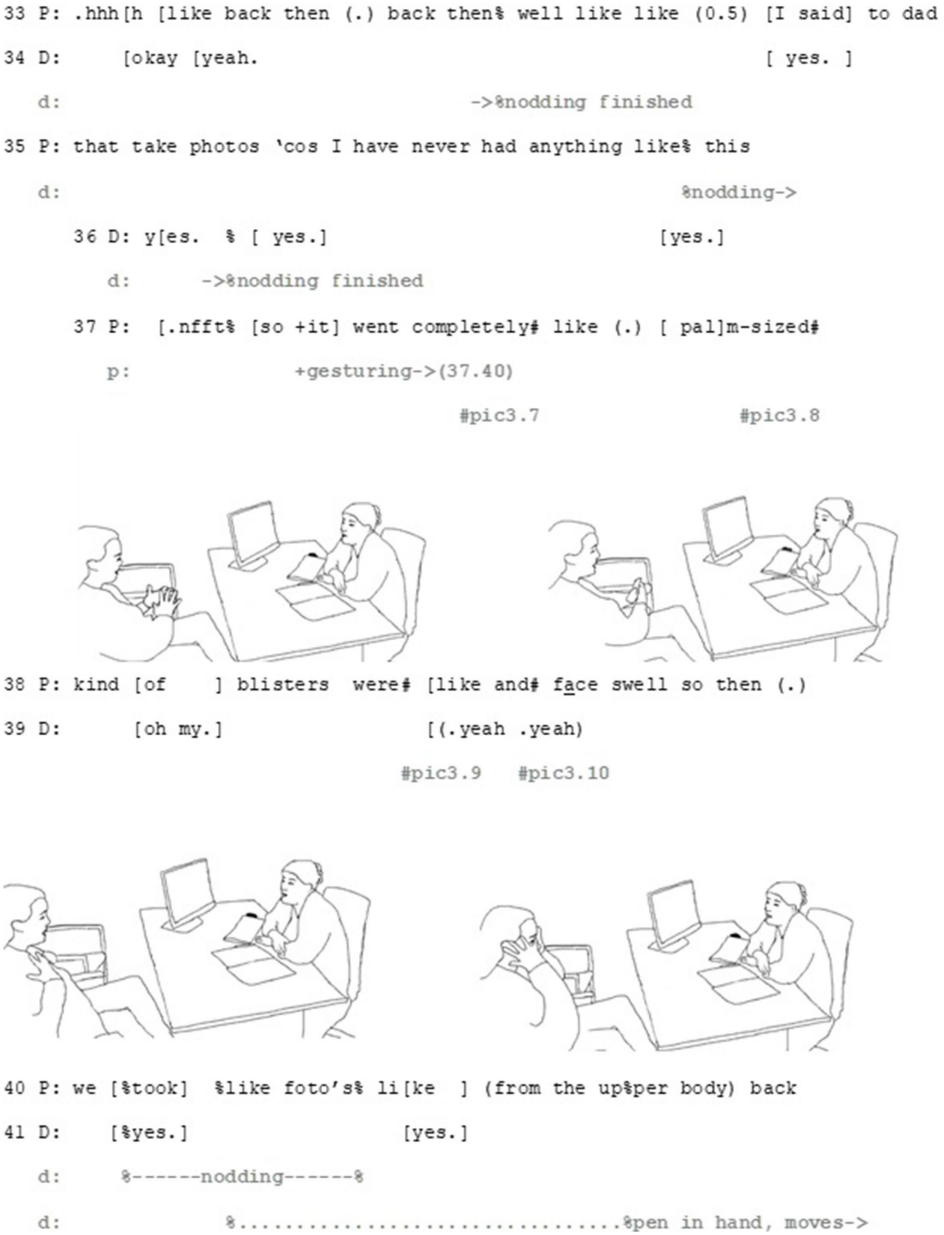
Extract 2—Part 2.

**Figure 7:**
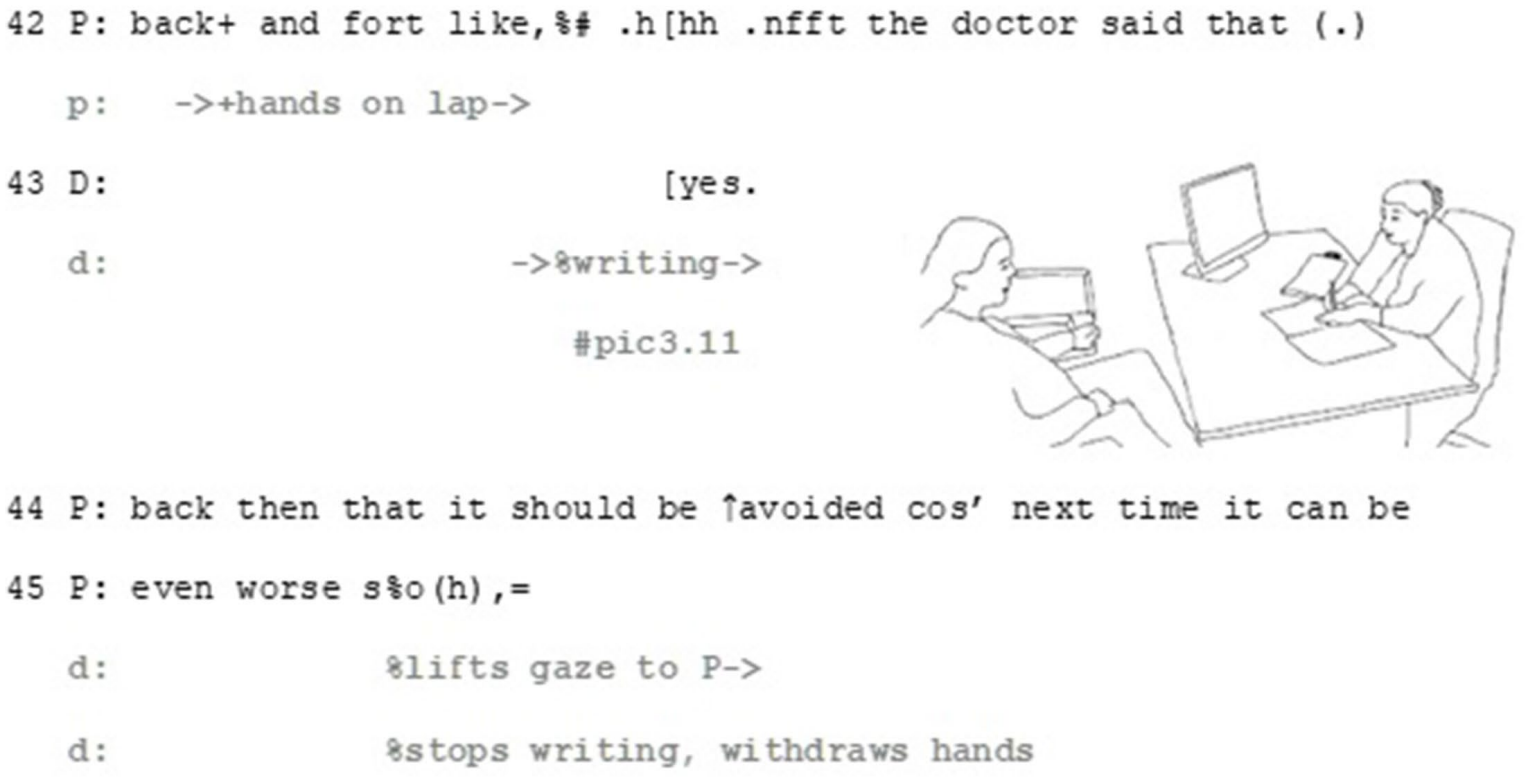
Extract 2—Part 2.

The overall activity, suggesting a medication, makes the patient’s deontic stance, that is, accepting or rejecting the suggestion, relevant. Furthermore, the doctor’s detailed description of symptoms of drug allergy makes it potentially a relevant topic for the patient to address: since the participants have not discussed the patient’s drug allergies before, any information considering it would be new information for the doctor, which may, then, be relevant with regard the final choice of medication. This launches the patient to tell about her earlier experience of a penicillin-induced allergic reaction (Part 2) (Figures 5–7).

Epistemic, deontic and affective axes intertwine in the patient’s response. Through her multi-unit turn, the patient brings forward her understanding of her existing allergy as potentially relevant for the decision. Both the beginning and the end of the patient’s turn depict the relevance of deontic orientation of this turn (see, e.g., Levinson, 2012 on the importance of the beginning and Sacks, 1987 on the endings of turns). The turn starts with the Finnish particle *no* (*well*, line 21), which can be used to produce an action that is not straightforwardly aligning with the previous action (Sorjonen and Vepsäläinen, 2016, 258–265) and thus implicates a need to negotiate before making a decision. Thus, against the sequential projection of a turn that should be doing accepting (or rejecting) the doctor’s suggestion, this turn beginning makes it apparent that this alignment is dependent on evaluating the information brought forward by the patient.

The patient informs the doctor that she has a drug allergy to a specific penicillin (line 21–22), and treats it firstly, as something she can evaluate: the described rash symptoms match the patient’s experience. The patient works multimodally to make this matching apparent in her response by recycling similar gestures as the doctor does when describing the rash (see lines 13–15 and pic3.2 and pic3.3 for the doctor’s gestures and lines 37–40 and pic3.9 for the patient’s matching gesture). Secondly, by matching her gestures with those of the doctor, she also manages to build connection between the two epistemic domains: her experience with medications and the side-effects are similar to the ones the doctor has just described (see Goodwin, 2013 on earlier actions as *substrate*). By doing so, the patient positions herself epistemically as capable to identify the allergic rash, showing her recognition and understanding of what the doctor has just described.

The patient ends her turn with an upshot formulation on how she should abstain from using specific type of penicillin, a notion that is being supported by citing another medical professional (see Goffman, 1981, pp. 124–159, on footing and the differentiation of speaker roles). Thus, by citing another professional, the patient can produce strong epistemic and deontic statement (*it must be avoided*) while avoiding interfering the professional epistemic domain or displaying a strong personal commitment to the statement. The turn-ending *että* (*so,* line 45) with a continuing intonation also hints that some kind of conclusion should be made from what has been said (Koivisto and Voutilainen, 2016), offering the doctor the chance to evaluate it. Thus, the patient marks her earlier experience as potentially relevant and offers it to the doctor to take a stance on.

The patient’s turn also involves elements that make affective stance relevant. By making the affective axis relevant, in addition to explicating her distressing experience, the patient also manages to bring forward her perspective in a professional-lead, institutional situation, where her means for steering the agenda are limited. The patient achieves this by a multimodal gestalt of talk and gesture. The word choices of the story, namely *severe* (line 30), *palm-sized blisters* (line 37–38) and the extreme-case formulation *I thought that I would die* (line 32, Pomerantz, 1986) portray her experience with side effects as exceptionally intense and severe. These words are produced as part of multimodal gestalts, where their affective importance is drawn from their precise concordance with matching gestures: the word *palm-sized* with rubbing hands together (line 37, pic3.7 and pic3.8), *blisters* with rubbing the chest (line 38, pic3.9) and *face swell* with gesturing to the face with open palm (line 38, pic 3.10). Through these gestalts the patient manages to upgrade her affective stance. Right after the client has described the size of the blisters, the doctor aligns with the affective stance displayed by the patient, by uttering *oh my* (line 39). This minimal turn allows her to immediately respond to the patient’s experience empathetically in institutionally relevant, task-oriented way, which allow the patient to continue her story.

**Figure 8:**
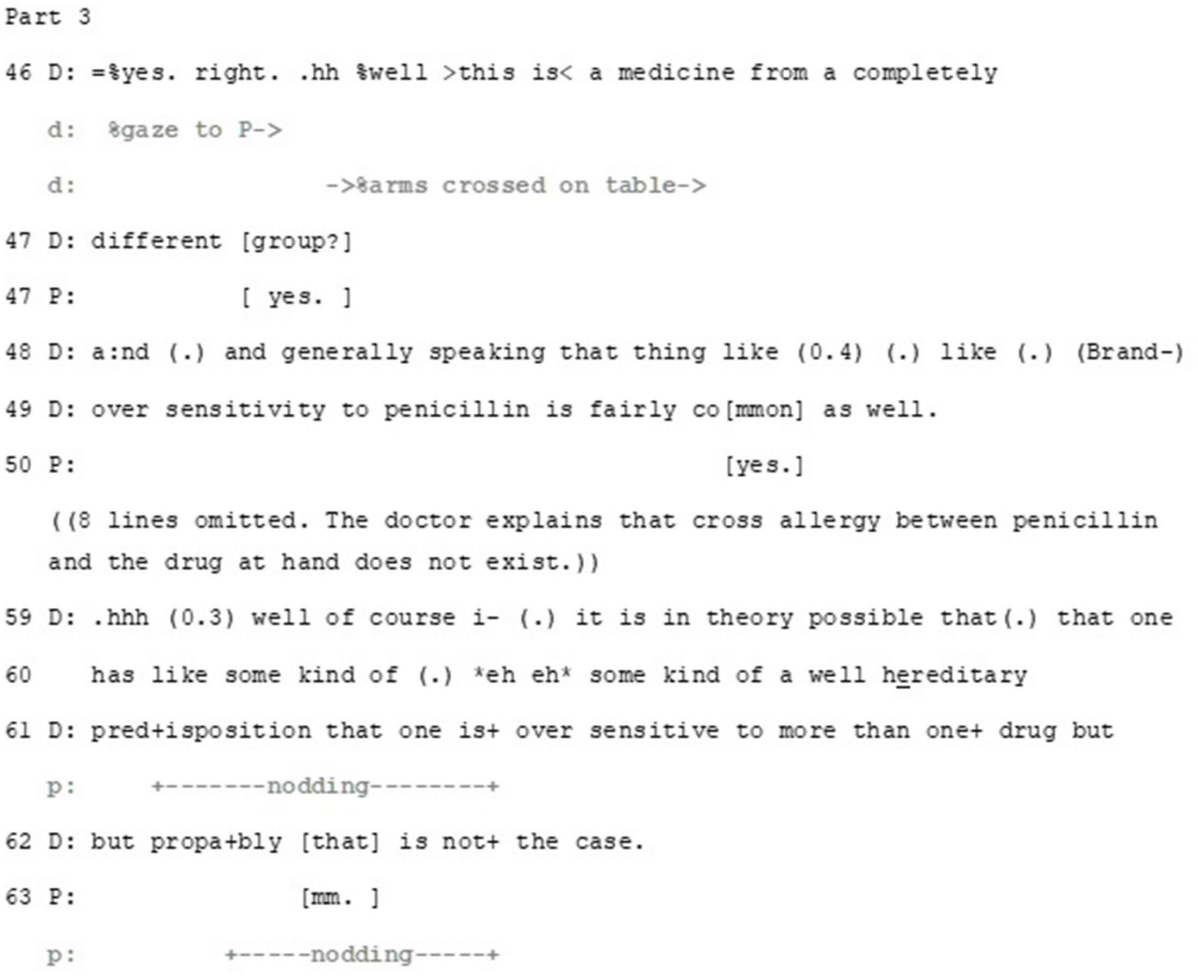
Extract 2—Part 3.

In her turn, the patient has managed to bring forward her epistemic stance (she treats her penicillin allergy as relevant here), deontic stance (should the penicillin allergy prove to be relevant by the doctor, the medicine should be changed) and affective stance (the earlier experience has been very distressing). The following parts of the extract depict how the doctor responds to the patient, first with a disaligning epistemic stance that also contains an affective dimension (Part 3) (Figure 8).

Overall, the doctor adopts high epistemic status in her stance that contrasts what the patient has said. The doctor first orients to the epistemic axis through providing information. This is initially done by an evaluation: there is a crucial difference between the medicine the patient is allergic to and the one to be prescribed (line 46). The *well* preface marks the contrast between what the patient just said and what the doctor is about the say and the *hän*-clitic marks the information as common knowledge (VISK §1,681). Furthermore, by using the expression “*a drug from a completely different group*” the doctor disaligns with the patient’s understanding of the penicillin allergy as being potentially relevant here by negating this. Thus, the doctor builds disalignment with what the patient has said and produces her epistemic authority on the topic. In so doing, the doctor also implicitly disaligns with the patient’s deontic stance that this issue should be taken into account in decision making about the medication. An affective orientation is also invested in the expression: the doctor’s extreme case formulation *‘from a completely different group’* can be heard as soothing the patient’s expressed worry about the potential side effects.

After elaborating the relationship between the two medicines (omitted) the doctor tells about the possibility of being allergic to multiple medicines (lines 59–62), framing this notion as *theoretical* and *unlikely*. The patient receives this information with nodding and a response token *mm*, thus aligning with the doctor’s more knowledgeable epistemic stance.

**Figure 9:**
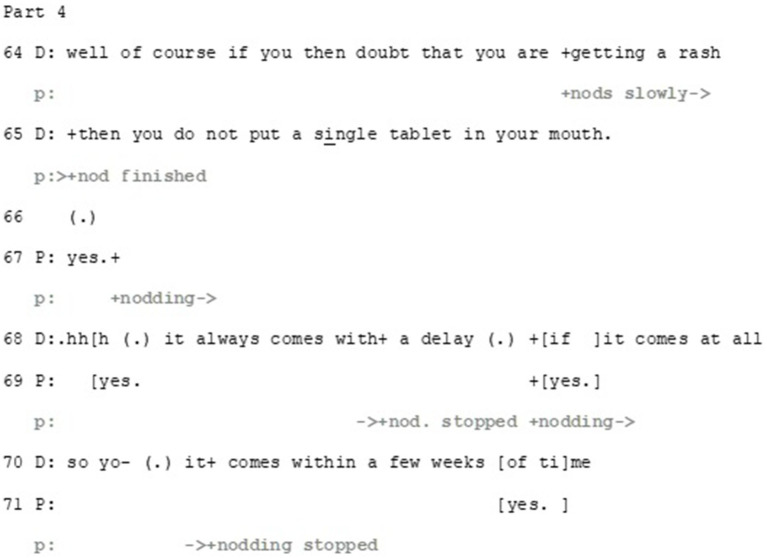
Extract 2—Part 4.

The doctor then proceeds to further address the deontic axis of the patient’s turn. Interestingly, this treatment of the deontic aspect also works to orient to the patient’s affect by displaying immediate interest in the patient’s well-being (Figure 9).

In spite of disaligning with the patient’s epistemic stance, the doctor aligns with the patient’s deontic stance—but with qualifications. Against the background of the unlikeliness of rash, the doctor states how even a *doubt* of rash warrants abstaining from the medication (line 64–65). It is noteworthy that here the doctor formulates the need to abstain from taking the medication as a strict order, enacting her epistemic and deontic status as a medical expert. Thus, while the doctor in essence disaligns with the patient’s epistemic and deontic stance (that the patient’s history of penicillin allergy should be a contra-indication to the suggested medication), she builds a hypothetical scenario that allows partial alignment. In addition, she provides the patient with necessary information on how to act if side-effects appear, thus aligning with patient’s epistemic positioning that she is capable to recognize the allergic rash.

The doctor also implies that despite the patient’s incorrect understanding about the medication, her previous experience, and the worry it brings, are being taken into account, thus aligning with the patient’s affective stance. Furthermore, by using an extreme-case formulation *do not put a single tablet in your mouth*, the doctor frames this issue as urgent and of high importance. Thus, she orients to a need to negotiate on deontic and epistemic aspects of proper treatment, and resorts to her medical expertise in taking the epistemic and deontic authority and responsibility in regards the result of the negotiation. In addition, the doctor manages the potential discrepancy by aligning with the patient’s affective stance, as with her extreme case formulation she conveys her understanding of the severeness of possible side effects that the patient has described in relation to her allergy.

Here it is apparent how the doctor disaligns with some of the patient’s stances and aligns with others: she corrects the patient’s notion about the potential relevancy of allergy to penicillin but aligns with the patient’s intense negative experience, achieving this in institutionally relevant minimal ways. Furthermore, regarding the deontics, she manages to both align and disalign with the patient’s hesitative stance on whether to accept the suggested medication or not. This is achieved by first providing information and reasoning why the patient’s idea about the penicillin allergy is not relevant in this case and then providing strong description of how to handle the conditional and unlikely event of getting allergic rash, which does align with the patient’s affective stance.

**Figure 10:**
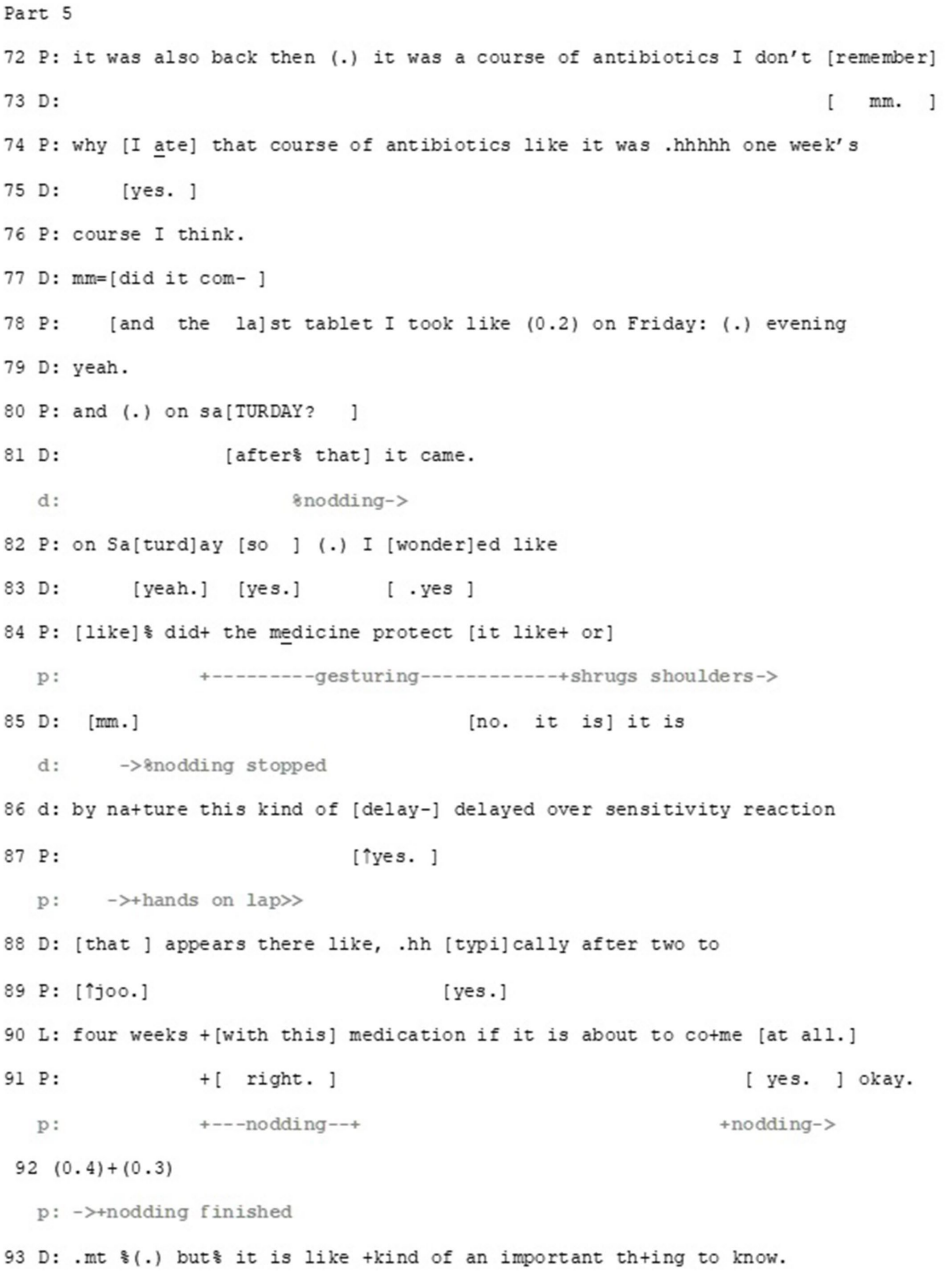
Extract 2—Part 5.

Despite the doctor having addressed all three stances, the patient expands her experience-telling and provides yet another epistemic evaluation of her previous rash (Part 5) (Figure 10).

The doctor has ended her turn with information about the timing of the occurrence of the side-effects (lines 68–70, part 4). The patient picks up on this (lines 72–82), displaying again the resemblance of her experience and what the doctor has described. At this point it is ambiguous whether the patient treats her experience still epistemically and deontically relevant regarding the decision-making: that is, if she continues her telling to show that she knows what a delayed effect might be and her ability to recognize one if necessary (thus aligning with the decision) or to make explicit that similar to the medicine to be prescribed also the earlier one caused delayed side-effects (thus resisting the decision).

Whether the patient is aligning or disaligning, the doctor expresses her understanding about what the patient is telling early on during the patient’s turn. As the patient is approaching the point in her story where she has taken the whole treatment, the doctor produces a potential end for the patient’s story (lines 80–81, note also that on line 77, she starts a yes-no question, which potentially would have carried the same pre-assumption). The doctor’s turn is built as a continuation to the patient’s turn (*I took the last pill on Saturday evening and*…) producing a grammatically compatible, collaborative completion to it (… *and then it came*). By this collaborative completion, the doctor shows that she not only understands what the patient is saying, but also that she *independently* knows this, once again producing epistemic authority.

After this, the patient brings forward another idea: the medication might have protected her from the rash (lines 82–84). Once again, the question-format enables her to present the idea without a strong commitment to it, supported by shrugging the shoulders. In her response, the doctor first orients to the epistemic axis by, again, disproving the idea (lines 85–90), followed by orientation to deontic axis, by repeating her earlier instructions (95–100), and finally to affective axis, by highlighting the immediacy of reacting to potential side-effects (*immediately*, lines 95 and 100). After this, the participants proceed to briefly discuss the interaction between the prescribed medicine and other medicines, before the doctor prints out the prescription.

## Discussion

In this article we have illustrated (1) how participants orient to multiple, even divergent stances as relevant at the same time and even in the same turn of talk, and (2) manage them with multimodal means to achieve closer alignment. In discussing patients’ ideas about their symptoms or future treatment, the participants orient to alternating between three axes of stance: deontic, epistemic and affective. They can emphasize the relevance of one over another, as in Example 1 where the epistemic stance was oriented to as focal, or they can address potential disalignment between some of the stances by orienting to the third, aligned one, as in Example 2 where the doctor softened the deontic and epistemic disalignment with the help of displaying aligning affective stance with the patient. The role of context obviously restricts or emphasizes the relevance of particular axes of stance, as in the clinical consultation in focus here, the institutional tasks of finding diagnosis and proper treatment for the patients’ ailment forms the main aim of the encounter, this way foregrounding the relevance of deontic and epistemic stances. As the epistemic and deontic statuses of the doctor are oriented to as higher, affective stance may afford more leeway for the patients to bring forward their own topics and concerns within the rather structured agenda of medical consultations. By evoking their own experiential knowledge and displaying affective stance they can interfere into the medical doctor-driven activity to introduce their own projects and manage their potentially disaligning stances concerning treatment decisions (see example 2).

We have also illustrated how the management of stances is multimodal. Epistemic and deontic stance may be conveyed for example through reported speech, and affective stance may be displayed both verbally, for example through extreme case formulations, and non-verbally, for example when enacting emotionally strenuous situations through gestures. The same multimodal turns can also be used to convey multiple stances at the same time, as in Example 2 where the patient imitated the doctor’s gestures in displaying her epistemic stance (in giving information about her previous rash) and her affective stance (by combining her extreme case formulations with the imitative gestures) (c.f. Mondada, 2018, on how multimodal actions may enable multiple temporal progressions in interaction).

Is there then some primacy between the management of stances? For example, should the stance(s) where the disalignment stands have a priority in the management process? This is simply too early to claim, as our data provides possibilities for both interpretation that this is the case (in extract 2, the doctor starts her multi-unit turn with epistemic orientation) and that this is not the case (already during the troubles-telling, the doctor uses *oh my* to do empathy). However, our data shows that being able to align on some axis may work as a lever to shift the disaligning stances on other axes toward closer alignment.

It is noteworthy that the management of stances entails subtle negotiation, turn by turn, where the displays of stance and also the balance of the different axes of stance are slightly modified, optimally resulting in reasonably sufficient intersubjective understanding and alignment which are necessary in order to proceed from one activity to another. In displays of stance, a certain cautiousness is observable: the original stance may be presented as open to change (example 1) or in a delicate way (example 2). This way, management of the simultaneous axes of stance appears to contribute both to maintaining the relationship of the participants and the progressivity of the consultation.

Our starting points have been the theoretical findings of Du Bois (2007), who has highlighted the intersubjective nature of stance and the interlocutors’ orientation to the alignment of stances, as well as the empirical work by Küttner (2019), Tai et al. (2022) and Sakita (2013, 2017), who have emphasized the negotiable and repairable nature of stance. Building on their observations and our own empirical work (see also Ruusuvuori and Peräkylä, 2009; Logren et al., 2019; Logren et al., 2020), we argue that in addition of straightforward *stance-taking,* a more flexible and situated *management of stance* was apparent in our cases. That is, in each action the participants took a stance in relation to one or more axes, but did it in ways that allowed the participants to adjust their stances and thus form alignment action-by-action (for example by expressing openness to change, delicacy or hypothetical scenarios). Furthermore, we have illustrated how the epistemic, deontic and affective axes of stance not only intertwine in interaction, but they have a crucial relationship with each other, into which the interlocutors observably orient to as relevant and consequential in relation to how their ongoing project can proceed. Nevertheless, the three axes are not, and do not need to be constantly parallel—as we have shown, their divergence *per se* seems not to be problematic for the interlocutors. Instead, divergence can actually work as a way to handle the disaligment of some of the stances: alignment in one stance seems to function to mitigate or even repair disalignment in the others.

We want to raise three points for methodological reflection. First, the examples we have provided here come from different types of sequential context: namely, asking a question at a transition between phases of the overall structure of the encounter (Example 1) and accepting/declining a suggestion during the decision-making phase (Example 2). Participants orient to achieving different tasks in different phases of a consultation which may shape both if and how patients bring forward different stances and how professionals respond to them. More robust, collection-based analysis of divergent stances in specific sequential environments followed by a systematic comparison of different contexts should provide steps for future theory building.

Second, our data are from a specific institutional context and as has been noted, this shapes the management of both epistemics, deontics and affect (Drew and Heritage, 1992; Heritage and Clayman, 2010; Jefferson and Lee, 1992; Robinson and Stivers, 2001; Ruusuvuori, 2005; Stivers, 2002). It has been suggested that the central task of institutional CA is describing *possible* social actions and their sequential and institutional conditions and the generalizability of these findings stem from comparison of the findings (Peräkylä, 2004). Thus, we do not claim generalizability of our findings but present potential questions to be asked from data from different context. As the management of divergent stances might be subordinate to the institutional task at hand, a question arises: how do interactants orient to different institutional relevancies when managing divergent stances? For instance, in psychotherapeutic contexts, the institutional task entails working with the patient’s emotions, and therefore the management of divergent stances can actualize in a very different form than in medical consultations. In addition, analyzing mundane conversations would provide even more insight on how divergent stances are managed without the institutional frameworks where specific deontic, epistemic or affective statuses may be inscribed in the institutional task. In short, more diverse data are needed to better understand the phenomenon.

And third, as the second extract exemplified, multimodal management of divergent stances can be messy, scattered and take a lot of time. Earlier research on, for example deontics, has focused on reasonably straightforward three-turn-structures (with clear benefits, such as producing robust theoretical formulations). However, when more than one stance is at stake, it seems that the action can disintegrate: resembling the tentacles of an octopus or a mycelium, one line of action can take one direction while others continue another way, just to compound at some unexpected moment. Studying complex phenomena such as the management of multiple simultaneous stances might then benefit from analytic strategies that expand beyond the sequence and follow the topical progression beyond the boundaries of an action sequence.

We conclude by stating that people do not just take a stance and stick with it: rather it seems that people modify their stances slightly as the interaction progresses, taking into account the stances displayed by their interlocutors. In each turn of talk, epistemic, deontic and affective stances are laminated, various stances are taken in the same turn, and the individual stances of the interlocutors are step by step shifted closer to each other—from disalignment to alignment. Therefore, interlocutors can eventually achieve at least partial alignment which they orient to as sufficient to enable them to proceed in the ongoing activity or task at hand without overt conflict or rupture in their relationship. Management of stances is thus not just a structural feature of interaction but may crucially influence the relationships of the interlocutors. It is observably relevant for the participants both in its local sequential context as well as in terms of the tasks they pursue together.

## Data Availability

The original contributions presented in the study are included in the article/Supplementary material, further inquiries can be directed to the corresponding author.

## Footnotes

^1^The authors want to thank Pinja Majava for producing the line drawings used in this article.

^2^VISK is the online version of the comprehensive Finnish grammar, edited by the Finnish Literature Society.
