# Supplementary material for: Management of divergent stances as a resource to maintain progressivity and social relationships
Source: Front Psychol. 2025 Mar 12;15:1436677. doi: 10.3389/fpsyg.2024.1436677 (PMC11937953; doi:10.3389/fpsyg.2024.1436677)
Supplement: Supplementary file 1 [file Data_Sheet_1.pdf]

Supplementary material for Logren, Ilomäki & Ruusuvuori (2025). Management of divergent stances as a resource to maintain progressivity and social relationships. *FrontPsychol*, doi: 10.3389/fpsyg.2024.1436677.

Appendix 1: Transcription symbols in the data extracts

[word]	Brackets: onset and offset of overlapping talk
=	Equals sign: contiguous utterances, second is latched immediately onto the first
(0.2)	Timed interval within or between utterances, measured in seconds and tenths of seconds
(.)	Interval of less than 0.2 s
wo:rd	Colon: extension of the sound or syllable
.	Full stop: falling intonation
,	Comma: continuing intonation
?	Question mark: rising intonation1
↑	Upward arrow: Rising pitch
wo-	Dash: abrupt cut-off
WORD	Capital letters: louder volume
<word>	Slower-paced talk than the surrounding talk
°word°	Degree signs: quieter volume
hh	Audible aspiration.
.hh	Audible inhalation
w(h)ord	Laughter
(----)	Lines in paranthesis: Unclear and unidentifiable talk
((word))	Text in parentheses: transcriber's comments
%	Percentile sign: The patient's bodily actions
+	Plus sign: The general practitioner's bodily actions
…	Line of full stops: Preparation of a bodily action
,,,	Line of commas: Retraction of a bodily action
#	Hash: The timing /location of the pictures
