# Supplementary material for: Management of divergent stances as a resource to maintain progressivity and social relationships
Source: Front Psychol. 2025 Mar 12;15:1436677. doi: 10.3389/fpsyg.2024.1436677 (PMC11937953; doi:10.3389/fpsyg.2024.1436677)
Supplement: Supplementary file 2 [file Data_Sheet_2.pdf]

Supplementary material for Logren, Ilomäki & Ruusuvuori (2025). Management of divergent stances as a resource to maintain progressivity and social relationships. *FrontPsychol*,

doi: 10.3389/fpsyg.2024.1436677.

Appendix 2: Original Finnish transcripts with English translations

Extract 1: Managing disaligning stances to achieve closer alignment

```
  d: >>stands up, moves towards the patient->
  p: >>gaze towards D->
 1 D: oisko sulla ollu tässä välissä kysymyksiä +%mieles+sä.
      would you at this point have questions in your mind.
   d:                                           %turns towards the Pat.->
   p:                                         ->+,,,,,,,+gaze forward->
 2     (0.4)%(0.2)
   d:    ->%standing facing the patient, back towards the camera->
 3 P: .hhh >no ei mulla kyllä varmaa< ettäh,
           well I quess I don’t have like,
 4    (0.3)
 5 D: ↑joo.
       yes.
 6 P: sillä% lailla tietysti on tuo mielee- .hh >mieleen on tullunna
      in that sense of course it has-           it has come to mind
   d:      %moves sideways, blocking the camera
 7 P: että< (0.8) kun kun (0.9) elokuussa sain sen viimesen (.)
     that         like like     in August I got that last (.)
 8 P: kolmannen (0.3) koronarokotteen että tuo[ta    ]  (0.3) että onkohan
      third           Covid  vaccination  like                like maybe

 9 D:                                          [↑mm-m.]
10 P: nyt sen jäläkeen tullu sitte vähä enemmän tätä touhua sitte että.
      after that there has been then a bit more of this hustle then like.
11 D: nii.
      yes.
12 P: .nii .että (.) oisko mahollista sieltä sitten (0.2)
       so   like     could it possibly from that then
13 P: [ johtuu (--)          ]
        come
14 D: [ .hh m-  mie  en  usko] että ne liittyy
            I- I don’t believe that they link
```

15 D: [siihen   koronarokotteeseen]

to that   covid vaccination

15 P: [>ei ne ehkä< siihen liitykkään] nii en minäkkään sitä usko

maybe they don’t link to that   yes I don’t believe that either

16 P: [kyllä et.]

like

17 D: [mm.   ]

18 P: .nii

yes

19 (0.4)

20 D: että: en: (.) en näkis sitä kovin todennäkö[senä]

so I wouldn´t I wouldn’t consider it very probable

21 P: [nii.]

yeah

22 D: et si[llä o]n mittään tekemistä tän asian kanssa.

that it has anything to do with this issue.

23 P: [.nii]

yeah

24 P: .joo

yes

25 (0.2)

26 P: kyl[lä.

yes

27 D: [.hhh hyvä.

good

Extract 2: Divergent epistemic, affective and deontic stances and their management

Part 1

d: >>gaze to P, arms crossed on table->

p: >>gaze to D, hands crossed on lap->

1 D: **#.hhhhh (.) >no sit eh%kä se %<tärkein asia tässä (0.3) niinku**

.hhhhh (.) >well then maby the< most important thing here (0.3) like

d: %,,,,,,%gaze to papers on table->

#pic3.1

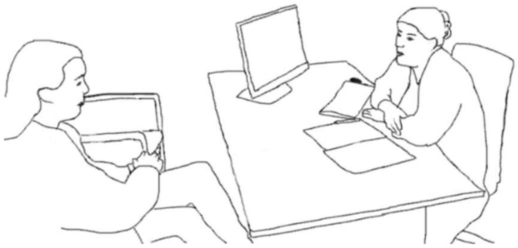

2 D: **alotusvaiheessa on että ehkä% noin %kymmenen prosenttia ihmisistä on**

at the beginning is that maybe about ten percent of people are

d: ->%......%gaze to P->

3 **on yliherkkiä tälle lääkkeelle.+=ihan samalla tavalla ku+ voi olla**

are over sensitive to this medicine.=exactly like one can be

p: +----------nods----------+

4 D: **asperiinille tai +peni[sil]liinille+ tai jolleki, .hhh muullekin**

to aspirin or penicillin or some other

5 P: **[mm]**

p: +------nods-------+

6 D: **lääkkeelle.**

medicine as well

7 **(0.8)**

8 D: **.mt ja se yliherkkyysihottuma on tavallisimmin (.) y-**

.mt and that oversensitivity rash is usually (.) o-

9 **yliherkkyys£r(h)eaktio£ on tavallisimmin ihottuma?**

over sensitivity r(h)eaction is usually rash?

10 D: **.hhh+h**

p: +nodding->

11 P: **joo.**

yes.

12 D: **eli se on semmonen+ vähän niin kun: (.) nokkosrokko >tai<**

so it is a bit like weld: (.) hives or

p: ->+stops nodding

13 >oikeestaan< (.) s- >semmonen vähän niin ku< vihurirokkomainen, (.)
actually t- >that kind of a bit like< rubella-like
14 +rokkomainen %ihot%#tuma+ jota voi #tulla, .hhhh (0.2) +niinku
pox-like rash which can occur .hhhh (0.2) like
d: %.....%gesturing on chest->
p:+--------nodding--------+ +nodding->
#pic3.2 #pic3.3

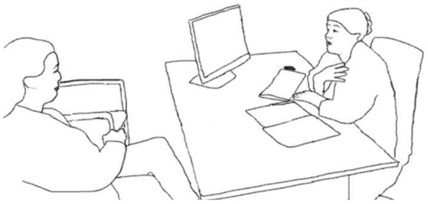

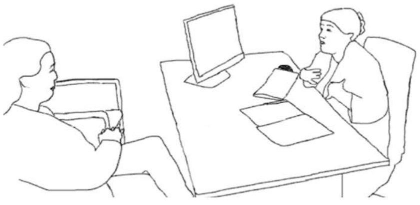

15 tähän, (.) vartalolle useimmin sen huomaa+ tästä
here to the body usually one spots it here
p: ->+nodding finished
16 D: rinta+kehän% [alu]eelta, (0.3) tai sitte+ raa%joihin.
at the chest area, (0.3) or then to the limbs.
d: ->%gesturing finished, moving hands>%arms on table->
17 P: [mm]
p: +-------------nodding--------------+
18 D: ja se kutisee.
and it itches.
19 (.)
20 D: +[siitä sen mone[sti huo]+ma[a.#
you can often tell it from that
Part 2
21 P: +[.hh [okei.]+ [n#o: tota: (0.2)
.hh okay well
p: +-------nodding----------+
22 P: Brand A-lääkeaine%al[lergia mulla% on?#
Allergy for Brand A I do have?
d: %turn to comp. %facing computer->
d: ->%..............%R hand on mouse
23 D: [joo.
yes.
#pic3.5

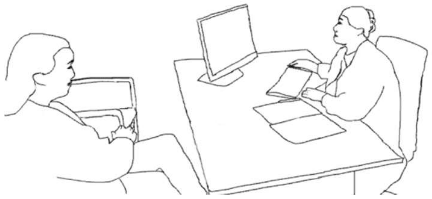

24 D: joo.
yes.
25 (.)

26 P: et%tä,
so
d: %click
27 (0.2)%(0.4)
d: %click
28 D: joo.
yes.
29 P: se on (.) >yhen kerran< sillon (.) ku se huomattiin ni se oli kyllä
it has the one time when we noticed it well then it was really
30 P: niin #raju% et%tä%
so severe that
d: %......%head turned to P->
d: %withdraws hand from the mouse->
31 D: joo.=
yes
32 P: =%(voi)% mä aatte%lin että mää kuolen siihen et%tä,#
(oh) I thought that I will die to this like
d: %----nodding----% %nodding->
d: ->%arms crossed on table->
#pic3.6

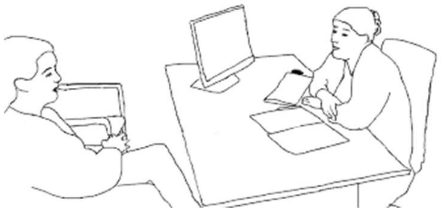

33 P: .hhh[h [et sillon (.) sillon% tota niin niin (0.5) [isälle] sanoin
like back then back then well like like I said to dad
34 D: [nii [just. [joo.]
okay yeah yes
d: ->%nodding finished
35 P: että ota kuvat että mul ei oo ikinä ollu tämmös%tä.
that take photos 'cos I have never had anything like this
d: %nodding->

36 D: j[oo. % [kyllä.] [joo.]
yes yes yes
d: ->%nodding finished
37 P: [.nfft% [et +se] meni ihan# semmoseks niinku (.) [käm]menen kokosia#
so it went completely like palm-sized
p: +gesturing->(37.40)
#pic3.7 #pic3.8

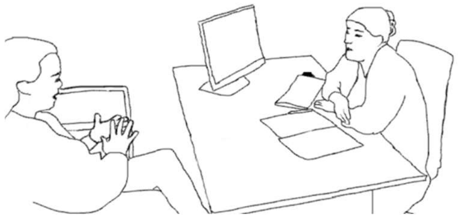
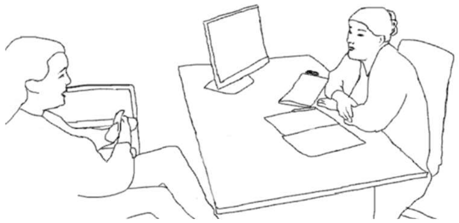

38 P: +semmo[sia] rakkuloita oli# [niinku ja #naama turpos +et sitte (.)
kind of blisters were like and face swell so then
39 D: [huh huh] [(.no .nii)
oh my yeah yeah
#pic3.9 #pic3.10

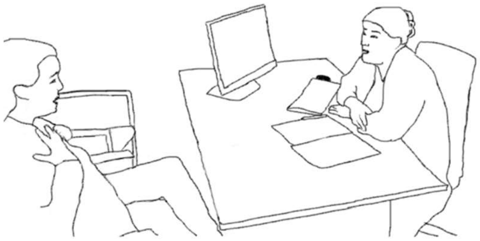
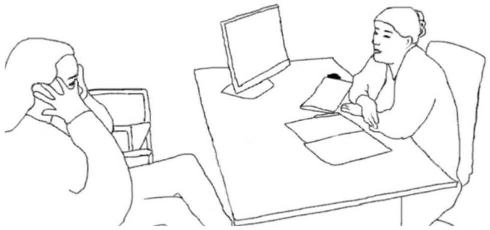

40 P: ote[%ttiin] +%niinku valokuvat% niin[ku] (yläke%hosta)
we took like foto’s like (from the upper body) back
41 D: [%joo.] [joo.]
yes yes
d: %-------nodding----------%
d: %...............................%pen in hand, moves->

42 P: ees+ sun taas sun että,%# .h[hh .nfft sitä sanoki lääkäri että (.)
back and fort like the doctor said that
p: ->+hands on lap->
43 D: [joo.
d: ->%writing->
#pic3.11

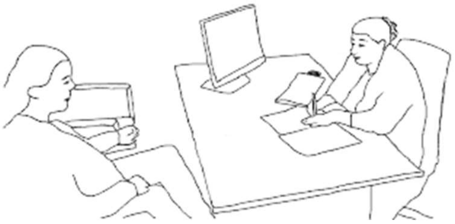

44 P: sillon et sitä pittää sitte ↑välttää
back then that it should be avoided c
45 P: kerralla vielä pahempi et%täh,=
even worse so
d: %lifts gaze to P->
d: %stops writing, withdraws hands

Part 3

46 D: =%nii. kyllä. .hh %no >tämähän o<ihan eri ryhmän lää[ke?]
yes. yes well this of course is a drug from a completely different group
d: %gaze to P->
d: ->%arms crossed on table->
47 P: [joo.]
yes
48 D: ja: (.) ja yleisesti ottaen se että (0.4) niinku (.) (a-)
and and generally speaking that thing like like (Bra-)
49 D: penisilliiniyliherkkyyshän on aika taval[linen] myös.
over sensitivity to penicillin is fairly common as well
50 P: [joo.]
yes
((8 lines ommitted. The doctor explains that cross allergy between penicillin and the drug at hand does not exist.))

59 D: .hhh (0.3) toki o(h)- (.) on teoriassa mahollista että (.) että on
well of course i- it is in theory possible that that one has
60 niinku semmonen (.) *öh eh* joku semmonen tuota perinnöllinen
like some kind of eh eh some kind of a like hereditary
61 D: altt+ius että on useemmalle+kin lääkkeelle yliherkkä mutta .hh (.)
predisposition that one is over sensitive to more that one drug but
p: +nodding +
62 D: mutta todennäkö+sesti [siitä] ei oo+ kyse.
but propably that is not the case

63 P: [mm.]
p: +------nodding------+

Part 4

64 D: no totta kai jos sä sitte epäilet että sulle +tulee ihottumaa niin
well of course if you then doubt that you are getting a rash
p: +nods slowly

65 D: +sit yhtään tablettia et laita suuhun.
then you do not put a single tablet in your mouth.
p:>nod finished

66 (.)

67 P: joo.+
yes.
p: +nodding->

68 D: .hh[h (.) se tulee aina viiväs+tetysti,(.) +[jos] se on tullakseen
it always comes with a delay if it comes at all

69 P: [kyllä. [joo.]
yes yes
p: ->+nod. stopped+nodding->

70 D: että su- (.) se+ tulee muutaman viikon [kulu]essa.
so yo- it comes within a few weeks

71 P: [joo.]
yes.
p: ->+nodding stopped

Part 5

72 P: seki oli sillon (.) se oli antibioottikuuri mä en [muista] mihinkä mä
it was also back then it was course of antibiotics I don't remember

73 D: [mm.]

74 P: sen [söin] sen antibioottikuurin et se oli .hhhhh viikon
why I ate that course of antibiotics like it was one week's

75 D: [joo.]

76 P: antibioottikuuriko se oli.
course I think

77 D: mm=[tuliks-]
did it com-

78 P: [ja viimi]sen tabletin otin niinkun (0.2) perjantai: (.) iltana.
and the last tablet I took like on Friday evening

```
79 D: nii.
      yeah

80 P: ja (.) lau[ANTAINA?   ]
      and    on Saturday
81 D:           [sen% jälkeen] tuli.
                after that it came
   d:               %nodding->
82 P: lauan[tai ]na [että  ] (.) si[tä mää] niinku ihmettelin
      on Saturday    so           I wondered  like
83 D:      [nii.]   [kyllä.]       [ .joo ]

84 P: [että]% suo+jasko se jotenkin se lääke [sitä että+ vai ]
       like   did the medicine protect it like or
   p:            +------------gesturing----------------+shrugs shoulders->
85 D:  [mm. ]                                [ei. vaan  se on] se on
                                              no         it is it is
   d:     ->%nodding stopped
86 D: luon+teeltaan tämmönen [viiväs-] viivästynyt yliherkkyysreaktio
      by nature this kind of delay-    delayed over sensitivity reaction
87 P:                        [↑ joo. ]
   p:   ->+hands on lap>>
88 D: [joka ] tulee siellä niinkun, .hhh [ tyy]pillisimmillään kahdesta
      that appears there  like            typically after two to
89 P: [↑joo.]                           [joo.]
90 D: neljään viikkoon  kuluessa +[tällä] lääk+keellä jos on tulla+k[seen.]
      four weeks             with this medication if it is about to come
91 P:                    +[just.]                              [joo. ] okei.
                          right                                yes     okay
   p:                    +---nodding--+                      +nodding->
 92 (0.4)+(0.3)
   p: ->+nodding finished
93 D: .mt %(.) mutta% se on niinku +semmonen tärkee as+ia tietää.
              but    it is like    kind of a  important thing to know
   d:     %.........%upper body turned to computer->
   p:                              +-------nodding----+
94 P: %mm.
   d:->%...
```

```
95 D: ja sitten% (.) otat heti yhteyttä jos +se [tulee] et sitte vaihetaan+
   and then    you contact us immediately if it comes and then we'll switch
   d: .........%orientation back to P
96 P:                                          [joo. ]
                                                 yes
   p:                                      +-----------nodding----------+
97 D: toiseen lääkkeeseen.
      to a different medication
98 P: joo.
      yes
99 D: ihan lennosta.
       immediately
00 P: joo.
       yes
```
